# Identification of novel inhibitors for TNFα, TNFR1 and TNFα-TNFR1 complex using pharmacophore-based approaches

**DOI:** 10.1186/s12967-019-1965-5

**Published:** 2019-07-02

**Authors:** Madhu Sudhana Saddala, Hu Huang

**Affiliations:** School of Medicine, Dept. Ophthalmology, Mason Eye Institute, University of Missouri, One Hospital Drive, MA102C, Columbia, MO 65212 USA

**Keywords:** TNFα, TNFR1, Pharmacophore modeling, Zinc database, Docking, ADMET

## Abstract

**Background:**

Tumor necrosis factor α (TNFα) is a multifunctional cytokine with a potent pro-inflammatory effect. It is a validated therapeutic target molecule for several disorders related to autoimmunity and inflammation. TNFα–TNF receptor-1 (TNFR1) signaling contributes to the pathological processes of these disorders. The current study is focused on finding novel small molecules that can directly bind to TNFα and/or TNFR1, preventing the interaction between TNFα or TNFR1, and regulating downstream signaling pathways.

**Methods:**

Cheminformatics pipeline (pharmacophore modeling, virtual screening, molecular docking and in silico ADMET analysis) was used to screen for novel TNFα and TNFR1 inhibitors in the Zinc database. The pharmacophore-based models were generated to screen for the best drug like compounds in the Zinc database.

**Results:**

The 39, 37 and 45 best hit molecules were mapped with the core pharmacophore features of TNFα, TNFR1, and the TNFα–TNFR1 complex respectively. They were further evaluated by molecular docking, protein–ligand interactions and in silico ADMET studies. The molecular docking analysis revealed the binding energies of TNFα, TNFR1 and the TNFα–TNFR1 complex, the basis of which was used to select the top five best binding energy compounds. Furthermore, in silico ADMET studies clearly revealed that all 15 compounds (ZINC09609430, ZINC49467549, ZINC13113075, ZINC39907639, ZINC25251930, ZINC02968981, ZINC09544246, ZINC58047088, ZINC72021182, ZINC08704414, ZINC05462670, ZINC35681945, ZINC23553920, ZINC05328058, and ZINC17206695) satisfied the Lipinski rule of five and had no toxicity.

**Conclusions:**

The new selective TNFα, TNFR1 and TNFα–TNFR1 complex inhibitors can serve as anti-inflammatory agents and are promising candidates for further research.

**Electronic supplementary material:**

The online version of this article (10.1186/s12967-019-1965-5) contains supplementary material, which is available to authorized users.

## Background

Tumor necrosis factor α (TNFα) is a cytokine secreted by macrophages in response to septic shock, inflammatory agents and cachexia. TNFα plays a key role in the immune system and cell death (e.g., apoptosis and necrosis) [1]. TNFα is involved in a number of autoimmune diseases, including psoriasis, inflammatory bowel disease, rheumatoid arthritis, systemic sclerosis, systemic lupus erythematosus, multiple sclerosis, diabetes and ankylosing spondylitis [2]. Since TNFα is an important mediator in infections and tumors, a series of biological agents targeted to TNFα has been developed for the treatment of cancer and autoimmunity [3].

TNFα contributes to the pathogenesis of inflammatory, edematous, neovascular, and neurodegenerative diseases of the eye [4]. Injection of TNFα into animal eyes induces breakdown of the blood–retina barrier [5]. Furthermore, increased levels of TNFα and TNF-receptors (TNFRs) have been found in the serum of humans with uveitis. Upregulation of TNFα expression has been shown in keratocytes of patients with rheumatoid corneal ulcerations [6]. Moreover, there is increasing evidence of TNFα involvement in the pathogenesis of experimental retinal neovascularization, proliferative vitreoretinopathy, and macular edema [7]. In an in vivo animal model of retinal injury, Berger et al. [8] showed that TNFα played a deleterious role in ischemia–reperfusion injury. Direct neutralization of this cytokine partially preserved retinal function [8]. The diverse characteristics of TNFα were attributed in part to the timing of its expression after injury. Nagineni et al. [9] demonstrated that inflammatory cytokines, including interleukin 1 beta (IL-1β), interferon gamma (IFN-γ) and TNFα, increased the secretion of vascular endothelial growth factor (VEGF)-A and -C by human retinal pigment epithelial (RPE) cells and choroidal fibroblasts, with VEGF being the most important factor for initiating pathological ocular neovascularization [9]. TNFα is crucial for the pathogenesis of diabetic retinopathy in rodents, and its pharmacological blockade leads to the inhibition of retinal cell death [10, 11].

A variety of TNFα antagonists, including infliximab, etanercept, adalimumab, certolizumab and glolimumab, were developed for therapeutic applications [12]. However, these biological therapies exhibited inevitable weaknesses, such as risk of infection, high cost, and the requirement for intravenous injections. By contrast, small molecule inhibitors are relatively cheaper and can be taken orally. Therefore, the identification of small molecules that can inhibit TNFα-regulated pathways is a promising research area that has lately received much attention.

Therefore, in the present study we used cheminformatics as part of the pipeline [pharmacophore modeling, virtual screening, molecular docking and in silico ADMET (absorption, distribution, metabolism, excretion and toxicity) analysis] to screen for novel, safe TNFα and TNFR1 inhibitors from the publicly available Zinc database.

## Methods

### Preparation of target proteins

We took the crystal structures of the target proteins: TNFα (2AZ5) with resolution 2.1 Å [13] and TNFR1 (1EXT) with resolution 1.85 Å, from the PDB (https://www.rcsb.org/). The TNFα–TNFR1 complex protein was downloaded from a public web site (http://www.cbligand.org/downloads/TNF_TNFR1.pdb). We removed all the hetero atoms and crystal water molecules from the target proteins and minimized the energy.

### Active site prediction

The active sites of TNFα, TNFR1 and the TNFα–TNFR1 complex were predicted using the CASTp (Computed Atlas of Surface Topography of proteins) server (http://sts.bioe.uic.edu/castp/index.html?2pk9). CASTp measures and identifies pockets and pocket mouth openings, in addition to the cavities. We uploaded the target proteins as input to predict the ligand binding sites. The CASTp server predicted the key amino acids for binding interactions to the inhibitors [14].

### Target-ligand pharmacophore generation

The structure-based pharmacophore technique can be used to advance the drug development process. For pharmacophore modeling, we selected three PDB (protein data bank) structures, i.e. TNFα (2AZ5), TNFR1 (1EXT), and the TNFα-TNFR1 complex (modelled protein) and their inhibitors (default inhibitor: 307), physcion-8-glucoside (ZINC33832439), Erythrosine B (ZINC08214556). The structure of the TNFα-307 complex was used as the fundamental of the pharmacophore modelling while TNFR1 with the physcion-8-glucoside and TNFα-TNFR1 complex with Erythrosine B complexes were used for pharmacophore design. ZINCPharmer (http://zincpharmer.csb.pitt.edu) is an online interface for searching the purchasable compounds of the Zinc database using the Pharmer pharmacophore search technology. ZINCPharmer can automatically extract a set of pharmacophore features from the molecular structure. Each feature comprises the feature type (hydrophobic, hydrogen bond donor/acceptor, positive/negative ion or aromatic), a position, and a search radius [15]. We provided both a receptor and bound-ligand structures, and ZINCPharmer is automatically identified an interaction pharmacophore. All possible pharmacophore features on the ligand were computed; however, only those that were within a distance cutoff of complimentary features on the receptor are enabled. We modified the pharmacophore features for TNFα, TNFR1 and the TNF-TNFR1 complexes and set parameters such as the hydrogen bond acceptors (HBA)/donors (HBD) are within 4 Å, charged and aromatic features are within 5 Å of the receptor.

### Pharmacophore based virtual screening

The modelled pharmacophores features were used as query features for screening for small molecules against the Zinc purchasable compound database. The TNFα has three aromatic spheres, two hydrophobic spheres, and one HBA spheres; TNFR1 has two aromatic spheres, one hydrophobic sphere, one HBD and one HBA spheres; TNF–TNFR1 has two aromatic spheres, one hydrophobic sphere, one HBD and one HBA spheres. Each pharmacophore model feature searched on Zinc purchasable compounds to get the hits based on matched features. The TNFα pharmacophore model obtained 39 hits, while TNFR1 had 37 and the TNF–TNFR1 complex had 45 hits. These hits were used for the docking studies.

### Docking simulation

Molecular docking studies were carried out with the AutoDock 4.2 in PyRx Virtual Screening Tool, which was used to generate the docking key files. Experiential free energy utility and Lamarckian genetic algorithm (LGA) with the following settings: a maximum of 2,500,000 energy evaluations, a preliminary population of 150 randomly placed individuals, a maximum of 27,000 generations, a transmutation rate of 0.02, and a crossover velocity of 0.8, along with an exclusiveness rate (numeral of top individuals to endure to the next generation) of one were designed for docking. The supposed Solis and Wets law was useful to a maximum of 300 iterations for each look for confined search. Default principles were thought to be designed for all the parameters not previously mentioned.

### Chemical analysis of drug-likeness

The drug-likeness properties were analyzed using MolSoft Drug-Likeness explorer (http://www.molsoft.com/mprop/) and the FAF-Drugs4 server (http://fafdrugs4.mti.univ-paris-diderot.fr/). Drug-likeness was indicated by the Lipinski “Rule of 5’’ [16]. Drug likeness can be described as a complex balance of various molecular properties and structural features, that determine whether a molecule is a drug or a non-drug. In accordance with the Lipinski “Rule of 5’’ a compound has a lot of possible elected membrane permeability and merely captivated through the body, if its relative molecular mass is a less than 500, its lipophilicity, expressed as an amount referred to as *LogP* is a less than five, the number of groups within the compound that may give hydrogen atoms to hydrogen bonds is a less than five, and the number of group that may settle for hydrogen atoms to make hydrogen bonds is a less than 10 [17].

### Prediction of physicochemical descriptors and ADMET parameters

The physicochemical profiles of lead compounds can increase the quality of clinical candidates [18]. The individual consideration of ADMET behaviors in the early stages of drug discovery have decreased the fraction of global pharmacokinetics related to failures in later phases of development. ADMET parameters of the best 15 compounds were predicted by SwissADME tools [19]. SwissADME predicts BBB (blood brain barrier) penetration and GI (gastro intestine) absorption by BOILED-Egg method [20]. It classified compounds as targets of *p*-glycoprotein (*p*-gp) efflux, inhibitors of cytochrome P450 enzymes CYP2C9, CYP2C19, CYP2D6 and CYP3A4 and substrates for metabolism by CYP2D6 and CYP3A4. It has predicted drug likeness by Lipinski, Ghose, Veber, Egan, Muegge methods and medicinal chemistry parameters by the Pan-Assay Interference Compounds (PAINS), Brenk methods and other parameters.

## Results

### Cheminformatics pipeline

**Fig. 1:**
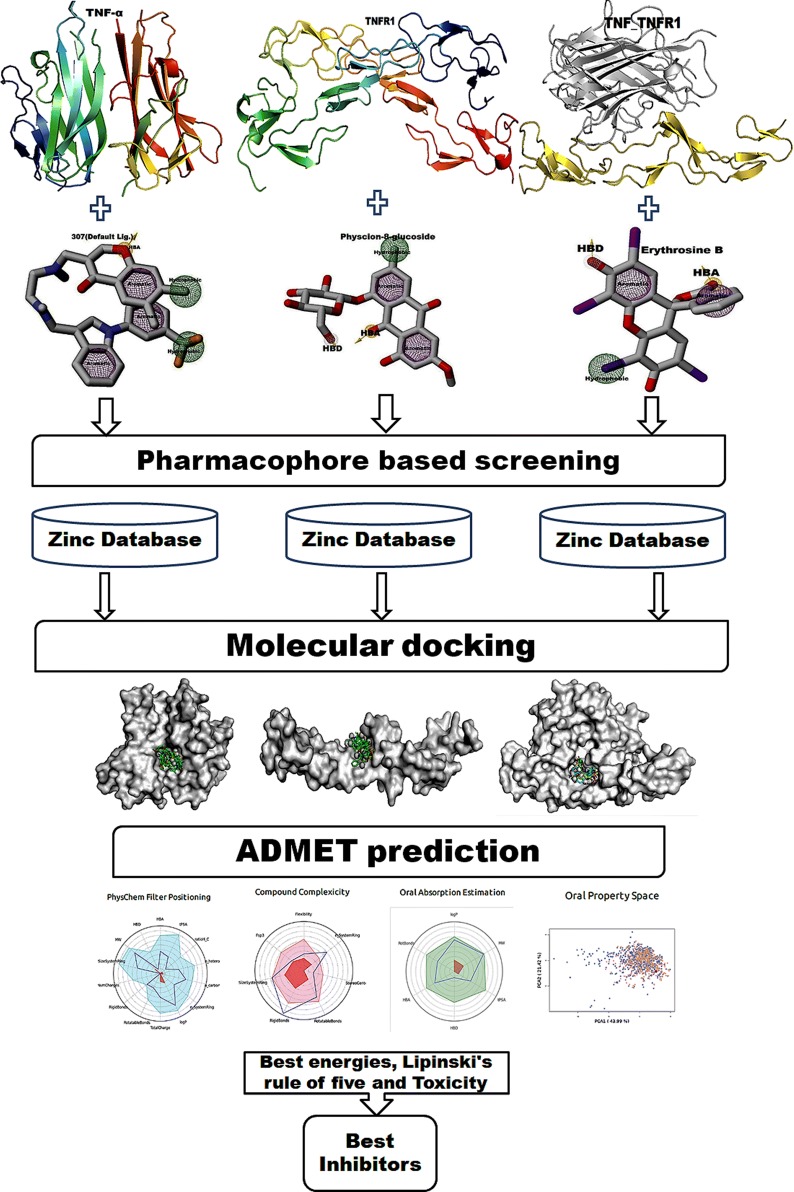
The detailed work flow of the present study. The cheminformatics part of the pipeline part of the pipeline indicates pharmacophore modeling, virtual screening, molecular docking and in silico ADMET analysis

TNFα is a cell signaling protein (cytokine) involved in systemic inflammation and is one of the cytokines that comprise the acute phase reaction. TNFα is produced chiefly by activated macrophages, although it can be produced by many other cell types. It is associated with a variety of important physiological processes and pathological conditions [21]. To control the adverse effects of TNFα, the current efforts have focused on blocking TNFα binding to its receptor. The overview flow chart of the cheminformatics pipeline for the present study is shown in Fig. 1.

### Target proteins preparation

The crystal structures of TNFα with a small molecule inhibitor (307) (2AZ5) [13] with resolution 2.1 Å, R-value free 0.278, and R-value work 0.220 and an extracellular domain of the 55 kda TNFR1 (1EXT) with resolution 1.85 Å, and R-value free 0.243, R-value work 0.203 were downloaded from the protein data bank (PDB) (https://www.rcsb.org/) [22]. The TNF α–TNFR1 protein complex was downloaded from Chen et al. [23] public web site (http://www.cbligand.org/downloads/TNF_TNFR1.pdb). All hetero atoms and crystal water molecules were removed from target proteins. We performed energy minimization by using the AutoDock Vina tool [24] with the following energy minimization parameters: a UFF (Universal force field) force field, a steepest descent optimization algorithm, 2000 steps for run, 1 step for update and an energy difference of less than 0.1. After energy minimization, target proteins were used for further analysis.

### Active site prediction

**Fig. 2:**
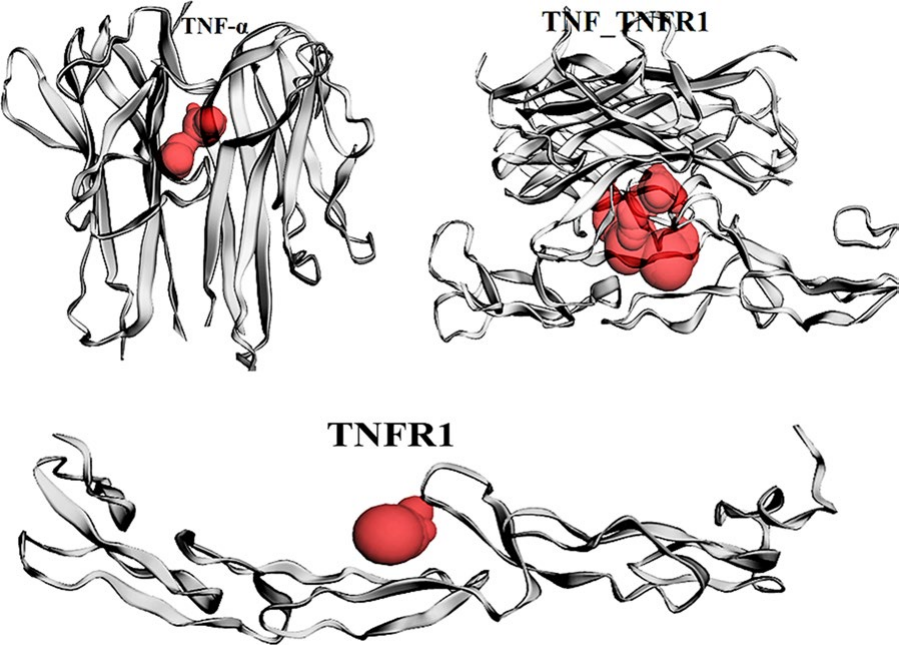
The active sites of TNF-α, TNFR1 and the TNF-α–TNFR1 complex. TNFα has a pocket ID of 2, an area of 104.507 and a volume of 35.048. The TNFR1 has a pocket ID of 2, an area of 24.807 and a volume of 26.187. The TNFα–TNFR1 complex has a pocket ID of 3, an area of 521.964 and a volume of 26.187

The active amino acid sites of TNFα, TNFR1 and the TNFα–TNFR1 complex were predicted using the CASTp server. CASTp identified pockets, pocket mouth openings and the cavities of TNFα, TNFR1 and the TNFα–TNFR1 complex. TNFα had a pocket ID of 2, an area of 104.507 and a volume of 35.048. TNFR1 had a pocket ID-2, an area of 24.807 and a volume of 26.187. The TNFα–TNFR1 complex had a pocket ID of 3, an area of 521.964 and a volume of 26.187. The CASTp server predicted the binding site amino acids of TNFα (Val91, Asn92, Leu93, Phe124 of chain-A, His15, Val17, Ala18, Pro20, Arg32 Ala33, Asn34, Ala35, Phe144, Glu146, Ser147, Gly148, Gln149 and Val150 of chain-B) [14], TNFR1 (Ser74, Lys75, Arg77, Asn110 and Leu111 of chain-A), and the TNFα–TNFR1 complex (His15, Val17, Ala18, Pro20, Arg32, Ala33, Asn34, Ala35, Phe144, Ala145, Glu146, Ser147, Gly148, Gln149, Val150, and Tyr151 of chain-A, Thr77, His78, Thr79, Ser81, Pro-90, Val91, Asn92, Leu93, Ser95, Ile97, Phe124, Glu135, Ile136, and Asn137 of chain-B, Phe60, Leu71, Ser72, Cys73, Ser74, Lys75, Arg77, Gln82, Cys96 and Leu111 of chain-R for binding interactions to the inhibitors). The active sites of the three target proteins are presented in Fig. 2. These active site amino acids play vital roles in the pathological consequences of the dysregulated TNF-TNFR1 signaling pathway.

### Target-ligand pharmacophore generation

**Fig. 3:**
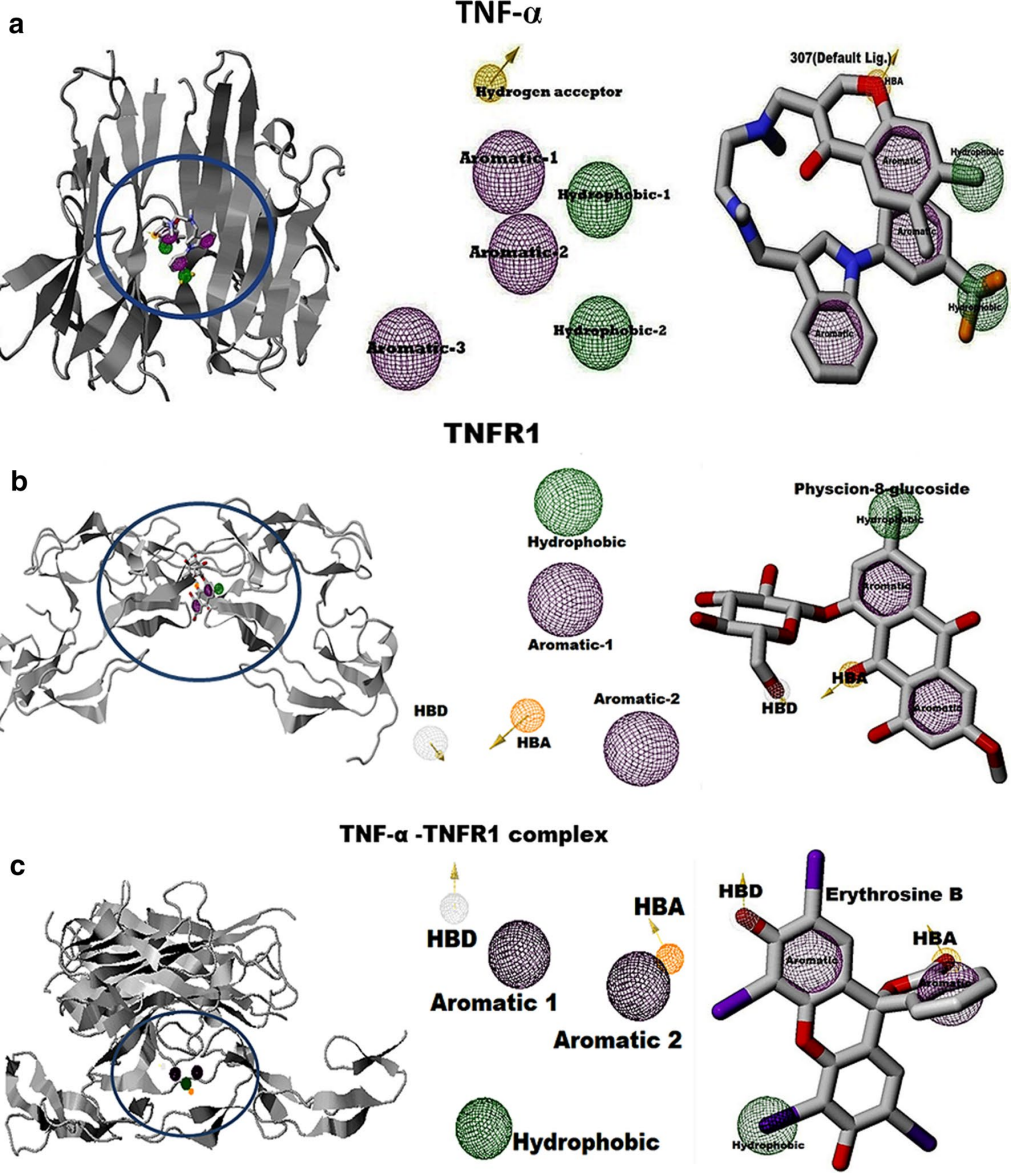
The pharmacophore features of TNF-α, TNFR1 and the TNF-α–TNFR1 complex. **a** The TNF-α pharmacophore contained six pharmacophoric features with default ligand (307), three aromatic spheres (pink), two hydrophobic spheres (green) and HBA (orange). **b** The TNFR1 pharmacophore contained five pharmacophoric features with Physcion-8-glucoside, one hydrophobic (green), two aromatic (pink), one HBA (yellow), HBD (white) features. **c** The TNF-α–TNFR1 complex pharmacophore contained five pharmacophoric features with Erythrosine B, one hydrophobic (green), two aromatic (pink), one HBA (yellow), HBD (white) features

The molecular binding process relies on several properties and features of the amino acids presenting in the active site [25]. According to the IUPAC (International Union of Pure and Applied Chemistry) definition, a pharmacophore is the ensemble of steric and electronic features that are necessary to ensure the optimal supramolecular interactions with a specific biological target structure and to trigger (or to block) its biological response. A pharmacophore model comprises several features organized in a specific 3D pattern. Each feature is typically represented as a sphere. Such pharmacophore features are typically used as queries to screen small molecule libraries of compounds [26]. The TNFα pharmacophore contains six pharmacophoric features with a default ligand 307 (Fig. 3a): three aromatic spheres (pink), two hydrophobic spheres (green) and one hydrogen bond acceptor (HBA) (orange) taken into consideration. The hydrogen acceptor (orange) has a 0.50 radius, along with x = − 17.85, y = 78.31, z = 34.03, θ = 50.782 and φ = 70.610. The aromatic sphere1 (pink) has a 1.10 radius, along with x = − 18.80, y = 76.42, z = 35.78, θ = 0.0 and φ = 0.0. The aromatic sphere2 (pink) has a 1.10 radius, along with x = − 16.78, y = 73.23, z = 34.14, θ = 0.0 and φ = 0.0. The aromatic sphere3 (pink) has a 1.10 radius, along with x = − 21.71, y = 71.83, z = 34.02, θ = 0.0 and φ = 0.0. The hydrophobic sphere1 (green) has a 1.00 radius, along with x = − 17.18, y = 75.75, z = 38.08, θ = 0.0 and φ = 0.0. The hydrophobic sphere1 (green) has a 1.00 radius, along with x = − 15.71, y = 71.21, z = 36.46, θ = 0.0 and φ = 0.0. The TNFR1 pharmacophore contains five pharmacophoric features: Physcion-8-glucoside (ZINC33832439), one hydrophobic (green), two aromatic (pink), one hydrogen bond acceptor (HBA, yellow), and hydrogen bond donor (HBD, white) features taken into consideration (Fig. 3b). The HBD sphere (white) has a 0.50 radius, along with x = 0.25, y = 36.09, z = − 10.12, θ = 138.098 and φ = − 78.856. The HBA sphere (yellow) has a 0.50 radius, along with x = 1.05, y = 32.22, z = − 10.32, θ = 137.203 and φ = − 102.266. The hydrophobic sphere (green) has a 1.00 radius, along with x = 4.71, y = 31.01, z = − 5.13, θ = 0.0 and φ = 0.0. The aromatic sphere1 (yellow-green sphere) has a 1.10 radius, along with x = 2.76, y = 31.26, z = − 7.24, θ = 0.0 and φ = 0.0. The other aromatic sphere2 (green) has a 1.10 radius, along with x = − 1.67, y = 30.08, z = − 9.17, θ = 0.0 and φ = 0.0. The TNFα-TNFR1 complex pharmacophore contains five pharmacophoric features with Erythrosine B (ZINC08214556), one hydrophobic (green), two aromatic (pink), one HBA (yellow), and HBD (white) features taken into consideration (Fig. 3c). The HBD sphere (white) has a 0.50 radius, along with x = 49.89, y = 20.00, z = 45.40, θ = 38.557 and φ = 108.405. The HBA sphere (yellow) has a 0.50 radius, along with x = 44.42, y = 14.29, z = 46.48, θ = 59.686 and φ = 78.543. The hydrophobic sphere (green) has a 1.00 radius, along with x = 51.17, y = 14.39, z = 40.97, θ = 0.0 and φ = 0.0. The aromatic sphere1 (yellow white sphere) has a 1.10 radius, along with x = 48.35, y = 17.79, z = 44.79, θ = 0.0 and φ = 0.0. The other aromatic sphere2 (green) has a 1.10 radius, along with x = 44.04, y = 16.11, z = 43.27, θ = 0.0 and φ = 0.0. All the pharmacophore features play a vital role in screening for the best lead compounds.

### Pharmacophore based virtual screening

The pharmacophore design models were used for screening molecules against the Zinc database (https://zinc.docking.org/). The pharmacophore features acted as query parameters along with hit reduction, hit screening and subset selection, which we set. For examples, Max Hits per Conformation was set to 10, Max Hits per Molecular was set to 1, Max Total Hits was set to maximum, Max RMSD (root mean square deviation) was set to 2, molecular weight (MW) was set to 300–500, rotatable bonds was set to 0–10. All the hit reduction and hit screening parameters were applied against the purchasable Zinc subset. Accordingly, the success of a virtual screening performance can be quantified by the enrichment factor (EF) and hit rate (HR) when the percentage of active compounds in the screening database is known. The TNFα pharmacophore model obtained 39 hits, TNFR1 had 37 and TNF-TNFR1 complex had 45 hits. The pharmacophore hits were mapped with the pharmacophore models. All the hit molecules were submitted to molecular docking studies.

### Target proteins and small compounds docking simulation

**Fig. 4:**
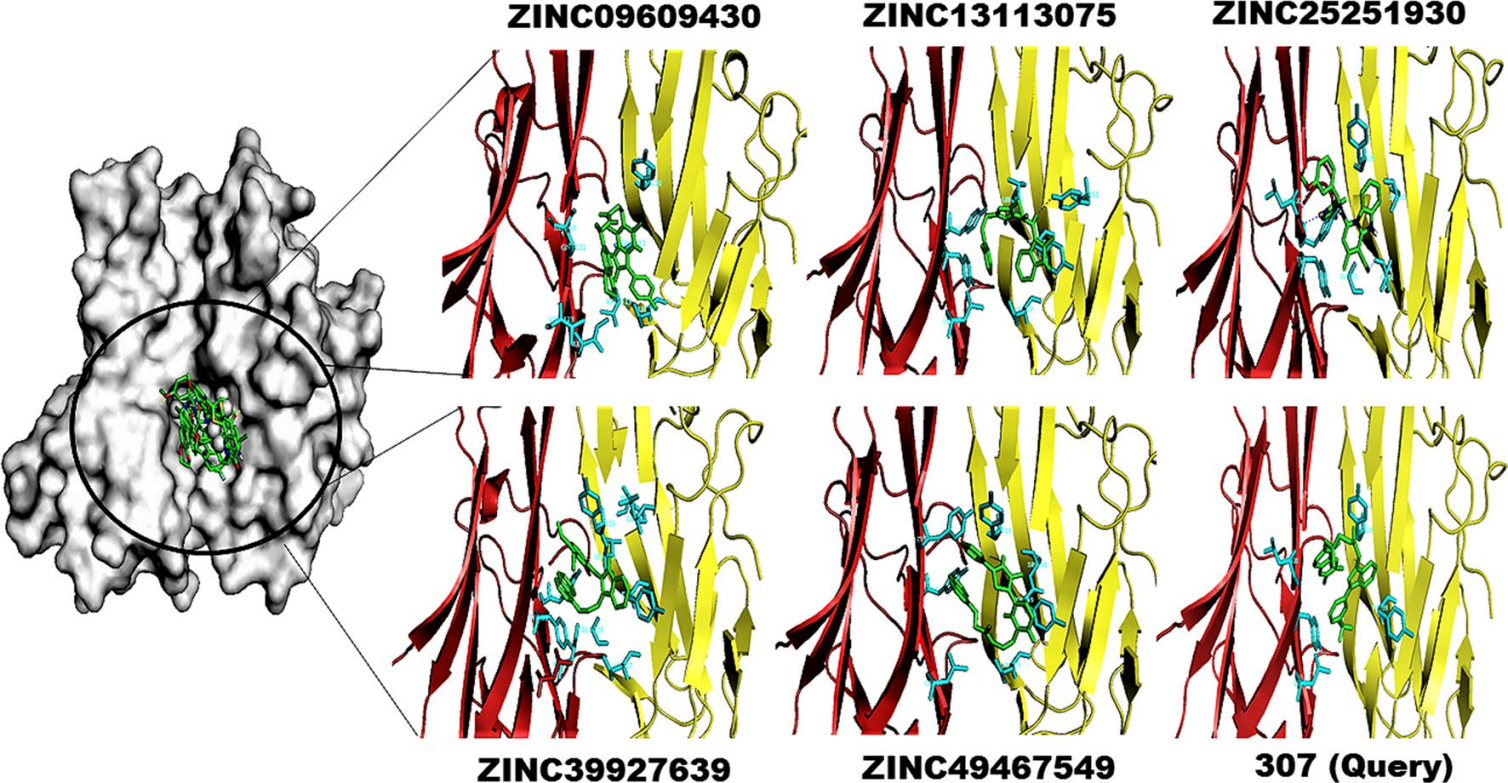
The top five ligand molecular poses of docking protein–ligand interaction analysis of TNFα along with docking functional residues. The names of these compounds are ZINC09609430, ZINC49467549, ZINC13113075, ZINC39907639 and ZINC25251930

Protein–ligand docking is the generally used docking algorithm. It predicts the site of a ligand when it is bound to its protein [27]. Mostly docking algorithms can make many possible structures; thus, the means to score each structure is also required, to identify those of greatest interest. Docking was performed using the AutoDock in PyRx Virtual Screening tool [28, 29]. The hit molecules were docked into the active site of TNFα, TNFR1 and the TNFα–TNFR1 complex. Based on the binding conformation AutoDock generated binding energies for all molecules. Table 1 shows TNFα, TNF1 and the TNFα-TNF1 complex’s best top five small molecules’ ZINC IDs, popular name, SMILES (Simplified molecular-input line-entry system), binding energies, protein–ligand interaction residues, angles, distance between hydrogen bonds and number of hydrogen bonds. The ZINC09609430 molecule HN group interacts with one hydrogen bond to the Gly121 amino acid CH group with − 9.2 kcal/mol^−1^ binding energy. The ZINC49467549 molecule may interact with electrostatic or Van der Waal bonds with − 9.0 kcal/mol^−1^ binding energy to the Ile58, Leu120, Gly121, and Tyr151 active site amino acids of TNFα. The ZINC13113075 molecule OC group interacts with one hydrogen bond to the Tyr151 amino acid CO functional group with − 8.8 kcal/mol^−1^ binding energy. The ZINC39907639 molecule may interact electrostatic or Van der Waal bonds with − 8.5 kcal/mol^−1^ binding energy to the Ile58, Leu120, Gly121, and Tyr151 active site amino acids of TNFα. The ZINC25251930 molecule NC, NH and OC groups interacted with three hydrogen bonds to the Ile58, Leu120, Gly121, and Tyr151 amino acids CO, CO, and CN functional groups with − 8.1 kcal/mol^−1^ binding energy respectively. The 307 (default ligand) molecule may interact with electrostatic or Van der Waal bonds with − 6.8 kcal/mol^−1^ binding energy to the Ile58, Leu120, Gly121, and Tyr151 active site amino acids of TNFα (Fig. 4). Ile58, Leu120, Gly121, and Tyr151 are key residues for interacting with small molecules to inhibit TNFα trimer formation.

**Table 1:** The list of TNFα, TNFR1 and TNFα-TNFR1 complex and their best lead molecules interactions, binding energies, smiles, bond angles, bond lengths and number of hydrogen bonds

TNF-α							
Ids	Popular name	SMILES	Binding energy (ΔG) kcal/mol^−1^	Protein and ligands H-bond interactions	Angles (°)	Distance (Å)	No. of H-bonds
ZINC09609430	*N*-[1-(2,5-dioxabicyclo[4.4.0]deca-7,9,11-trien-8-yl)ethyl]-3-[7-(4-fluorophenyl)-2,4,8-trimethyl-1,5	Cc1c(c(n2c(n1)c(c(n2)C)c3ccc(cc3)F)C)CCC(=O)N[C@H](C)c4ccc5c(c4)OCCO5	− 9.2	Gly^121^ CH–HN	107.21	3.17	01
ZINC49467549	*N*-[2-[3-(4-methyl-2-phenyl-thiazol-5-yl)-6-oxo-pyridazin-1-yl]ethyl]-2-(1-naphthyloxy)acetamide	Cc1c(sc(n1)c2ccccc2)c3ccc(=O)n(n3)CCNC(=O)COc4cccc5c4cccc5	− 9.0	Ile^58^, Leu^120^, Gly^121^ and Tyr^151^	–	–	–
ZINC13113075	(2*R*)-2-[[5-[(1*S*)-1-(4-fluorophenoxy)ethyl]-1,3,4-oxadiazol-2-yl]sulfanyl]-1-(2-methyl-1H-indol-3-yl)	Cc1c(c2ccccc2[nH]1)C(=O)[C@@H](C)Sc3nnc(o3)[C@H](C)Oc4ccc(cc4)F	− 8.8	Tyr^151^ CO–OC	98.29	3.29	01
ZINC39907639	2-[(3,4-dimethoxyphenyl)methyl]-1-[2-(1-naphthyloxy)ethyl]benzimidazole	COc1ccc(cc1OC)Cc2nc3ccccc3n2CCOc4cccc5c4cccc5	− 8.5	Ile^58^, Leu^120^, Gly^121^ and Tyr^151^	–	–	–
ZINC25251930	1-[[(*R*)-(3-methoxyphenyl)-(1-methylimidazol-2-yl)methyl]amino]-3-methyl-pyrido[1,2-a]benzimidazole-4	cc1cc(n2c3ccccc3nc2c1C#N)N[C@H](c4cccc(c4)OC)c5nccn5C	− 8.1	Ile^58^ CO–NC	3.16	17.87	03
				Leu^120^ CO–NH	3.31	39.97	
				Gly^121^ CN–OC	2.99	111.96	
307 (Query)	6,7-dimethyl-3-[(methyl{2-[methyl({1-[3-(trifluoro methyl)phenyl]-1*H*-indol-3-yl}methyl)amino]ethyl}amino)methyl]-4*H*-chromen-4-one	Cn(ccn(c)cc1=c[n](c2=cc(=cc=c2)c(f)(f)f)c3=cc=cc=c13)cc4=coc5=cc(=c(c)c=c5c4=o)c	− 6.8	Ile^58^, Leu^120^, Gly^121^ and Tyr^151^	–	–	–

TNFR1							
Ids	Popular name	SMILES	Binding energy (ΔG) kcal/mol^−1^	Protein and ligands H-bond interactions	Angles (°)	Distance (Å)	No. of H-bonds
ZINC02968981	*N*-benzyl-2-[[2-(4-nitrophenyl)-[1, 2, 4]triazolo[1,5-c]quinazolin-5-yl]thio]acetamide	c1ccc(cc1)CNC(=O)CSc2nc3ccccc3c4n2nc(n4)c5ccc(cc5)[N+](=O)[O–]	− 10.1	Lys^75^ CO–ON Gln^82^ CO–ON Gln^82^ CN–ON Arg^104^ CN–OC Tyr^106^ CO–OC	112.42 97.99 101.92 109.87 149.80	3.25 3.35 3.16 2.84 3.20	05
ZINC09544246	2-[[5-(1*H*-indol-3-yl)-1,3,4-oxadiazol-2-yl]sulfanyl]-*N*-(3-sulfamoylphenyl)-acetamide	c1ccc2c(c1)c(c[nH]2)c3nnc(o3)SCC(=O)Nc4cccc(c4)S(=O)(=O)N	− 9.8	Glu^56^ CN–OC Glu^56^ CO–NH Ser^57^ CO–NH Ser^59^ CO–OC Cys^70^ CN–OC Cys^73^ CO–NH Ser^74^ CO–NH	167.25 106.92 119.86 124.31 109.30 150.85 105.01	2.93 2.06 3.34 2.90 3.06 3.24 3.19	07
ZINC58047088	*N*-[(4-fluorophenyl)methyl]-2-[(3-methyl-2-oxo-[1, 2, 4]triazino[2,3-c]quinazolin-6-yl)sulfanyl]acetami	Cc1c(=O)nc2c3ccccc3nc(n2n1)SCC(=O)NCc4ccc(cc4)F	− 9.5	Ser^74^ CO–NH Asn^110^ CO–NH	131.62 123.93	3.07 3.01	02
ZINC72021182	2-(4-chlorobenzoyl)-*N*-[2-[(2-hydroxybenzoyl)amino]ethyl]benzamide	c1ccc(c(c1)C(=O)c2ccc(cc2)Cl)C(=O)NCCNC(=O)c3ccccc3O	− 9.3	Arg^104^ CN–OH	85.81	2.98	01
ZINC08704414	2-(methylBLAHyl)sulfanyl-*N*-(*p*-tolylmethyl)propanamide	Cc1ccc(cc1)CNC(=O)[C@@H](C)Sc2nc3ccccc3c4n2nc(n4)C	− 9.1	Ser^74^ CO–NH Lys^75^ CO–NH Arg^77^ CN–OC Asn^110^ CO–NH	55.03 30.27 101.39 111.93	3.13 3.11 3.02 3.37	04
ZINC33832439 (query)	1-Hydroxy-3-methoxy-6-methyl-8-(((2*S*,3*R*,4*S*,5*S*,6*R*)-3,4,5-trihydroxy-6-(hydroxymethyl)tetrahydro-2H-pyran-2-yl)oxy)anthracene-9,10-dione	Cc1cc2c(c(c1)O[C@H]3[C@@H]([C@H]([C@@H]([C@H](O3)CO)O)O)O)C(=O)c4c(cc(cc4O)OC)C2=O	− 7.6	Arg^77^ CN–OC Cys^96^ CN-OH Cys^96^ CO–OH Arg^104^ CN—OH Arg^104^ CN–OH Arg^104^ CN—OH Tyr^106^ CO–OH Asn^110^ CO–OH	111.69 106.76 151.20 92.64 120.04 107.99 58.49	3.01 3.09 2.70 3.31 2.81 3.00 3.16	08

TNFα-TNFR1 complex							
Ids	Popular name	SMILES	Binding energy (ΔG) kcal/mol^−1^	Protein and ligands H-bond interactions	Angles (°)	Distance (Å)	No. of H-bonds
ZINC05462670	Chaetochromin B	C[C@@H]1[C@H](Oc2cc3c(c(cc(c3c4c5cc6c(c(c5c(cc4O)O)O)C(=O)[C@@H]([C@@H](O6)C)C)O)O)c(c2C1=*O)O)C*	− 10.0	Ser^74^ CO–OH Thr^94^ CO–OH Glu^109^ CO–OC Asn^110^ CO–OH	72.63 27.39 121.49 45.29	3.19 2.93 3.36 2.73	04
ZINC35681945	*N*2-[(2*R*)-2-(dimethylamino)-2-(2-methoxyphenyl)ethyl]-*N*4-(4-ethoxyphenyl)-5-nitro-pyrimidine-2,4,6-tr	CCOc1ccc(cc1)Nc2c(c(nc(n2)NC[C@@H](c3ccccc3OC)[NH+](C)C)N)[N+](=O)[O–]	− 9.7	Lys^75^ CO–NH Gln^82^ CO–ON Gln^82^ CN–ON Asp^93^ CO–NH Arg^104^ CN–OC Asn^110^ CO–NH	76.93 114.27 111.31 155.37 95.38 129.41	3.32 3.08 3.38 2.89 3.20 2.79	06
ZINC23553920	6-[[4-allyl-5-(2-methoxyphenyl)-1,2,4-triazol-3-yl]sulfanylmethyl]-*N′*-(2-ethylphenyl)-1,3,5-triazine	CCc1ccccc1Nc2nc(nc(n2)N)CSc3nnc(n3CC=C)c4ccccc4OC	− 9.5	Ser^74^ CO–OC Thr^94^ CO–NH Thr^94^ CO–NH	75.40 46.06 52.19	3.49 3.12 3.20	03
ZINC05328058	DNC011371	C[C@H]1CC(=O)c2c(cc3c(c2O)c(cc(c3c4c5cc6c(c(c5c(cc4O)O)O)C(=O)C[C@@H](O6)C)O)O)O1	− 8.9	Ser^74^ CO–OH Gln^82^ CO–OC Pro^90^ CO–OH Asn^92^ CN–OH Cys^96^ CN–OH	49.49 103.84 73.92 29.76 65.40	2.80 3.48 3.24 2.97 2.95	05
ZINC17206695	(2*Z*)-2-(2,5-dimethoxyphenyl)imino-6-[(3*R*,5*S*)-3,5-dimethyl-1-piperidyl]-5-nitro-1*H*-pyrimidin-4-amine	C[C@@H]1C[C@@H](CN(C1)c2c(c(nc(n2)Nc3cc(ccc3OC)OC)N)[N+](=O)[O–])C	− 8.5	Ser^74^ CO–NH Ser^74^ CO–NH Asn^93^ CO–NH Asn^110^ CN–ON Asn^110^ CN–NO Ser^147^ OC–OC	55.45 30.72 47.81 102.70 54.86	2.81 3.20 2.73 2.81 3.35	06
ZINC08214556 (query)	Erythrosine sodium (USP)	c1ccc2c(c1)C(=O)OC23c4cc(c(c(c4Oc5c3cc(c(c5I)O)I)I)O)I	− 7.2	Thr^94^ CO–OC Asn^110^ CN–OC	120.52 102.30	3.05 3.20	02

**Fig. 5:**
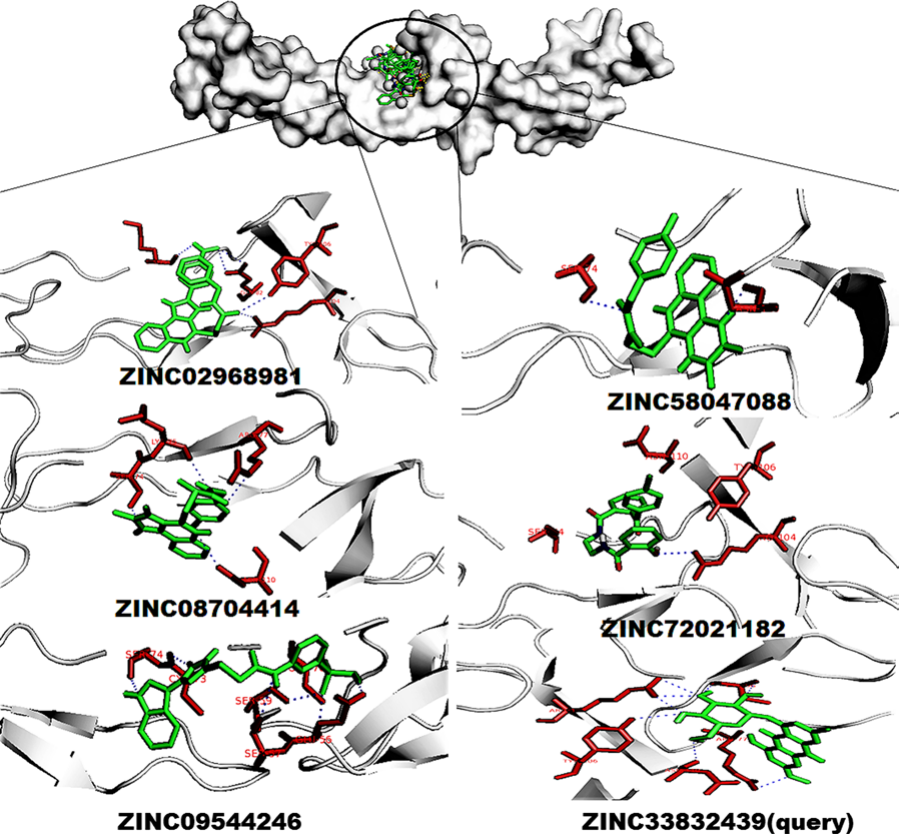
The top five ligand molecular poses of docking protein–ligand interaction analysis of TNFR1 along with docking functional residues. The names of these compounds are ZINC02968981, ZINC09544246, ZINC58047088, ZINC72021182 and ZINC08704414

The TNFR1 inhibitors, ZINC02968981 molecule ON, ON, ON, OC and OC groups interacted with five hydrogen bonds to the Lys75, Gln82, Gln82, Arg104 and Tyr106 amino acids CO, CO, CN, CN, and CO functional groups with − 10.1 kcal/mol^−1^ binding energy respectively. The ZINC02968981 molecule ON, ON, ON, OC and OC groups were interacted with five hydrogen bonds to the Lys75, Gln82, Gln82, Arg104 and Tyr106 amino acids CO, CO, CN, CN, and CO functional groups with − 10.1 kcal/mol^−1^ binding energy respectively. The ZINC09544246 molecule OC, NH, NH, OC, OC, NH and NH groups interacted with seven hydrogen bonds to the Glu56, Glu56, Ser57, Ser59, Cys70, Cys73 and Ser74 amino acid CN, CO, CO, CO, CN, CO and CO functional groups with − 9.8 kcal/mol^−1^ binding energy respectively. The ZINC58047088 molecule NH, and NH groups interacted with two hydrogen bonds to the Ser74 and Asn110 amino acid CO and CO functional groups with − 9.5 kcal/mol^−1^ binding energy respectively. The ZINC72021182 molecule OH group interacted with one hydrogen bond to the Arg104 amino acids CN functional group with − 9.3 kcal/mol^−1^ binding energy respectively. The ZINC08704414 molecule NH, NH, OC, OC, and NH groups interacted with four hydrogen bonds to the Ser74, Lys75, Agr77 and Asn110 amino acid CO, CO, CN, and CO functional groups with − 9.1 kcal/mol^−1^ binding energy respectively. The ZINC09544246 (Query) molecule OC, OH, OH, OH, OH, OH, OH and OH groups interacted with eight hydrogen bonds to the Arg77, Cys96, Cys96, Arg104, Arg104, Arg104, Tyr106 and Asn110 amino acid CN, CN, CO, CN, CN, CN, CO and CO functional groups with − 7.6 kcal/mol^−1^ binding energy respectively (Fig. 5).

**Fig. 6:**
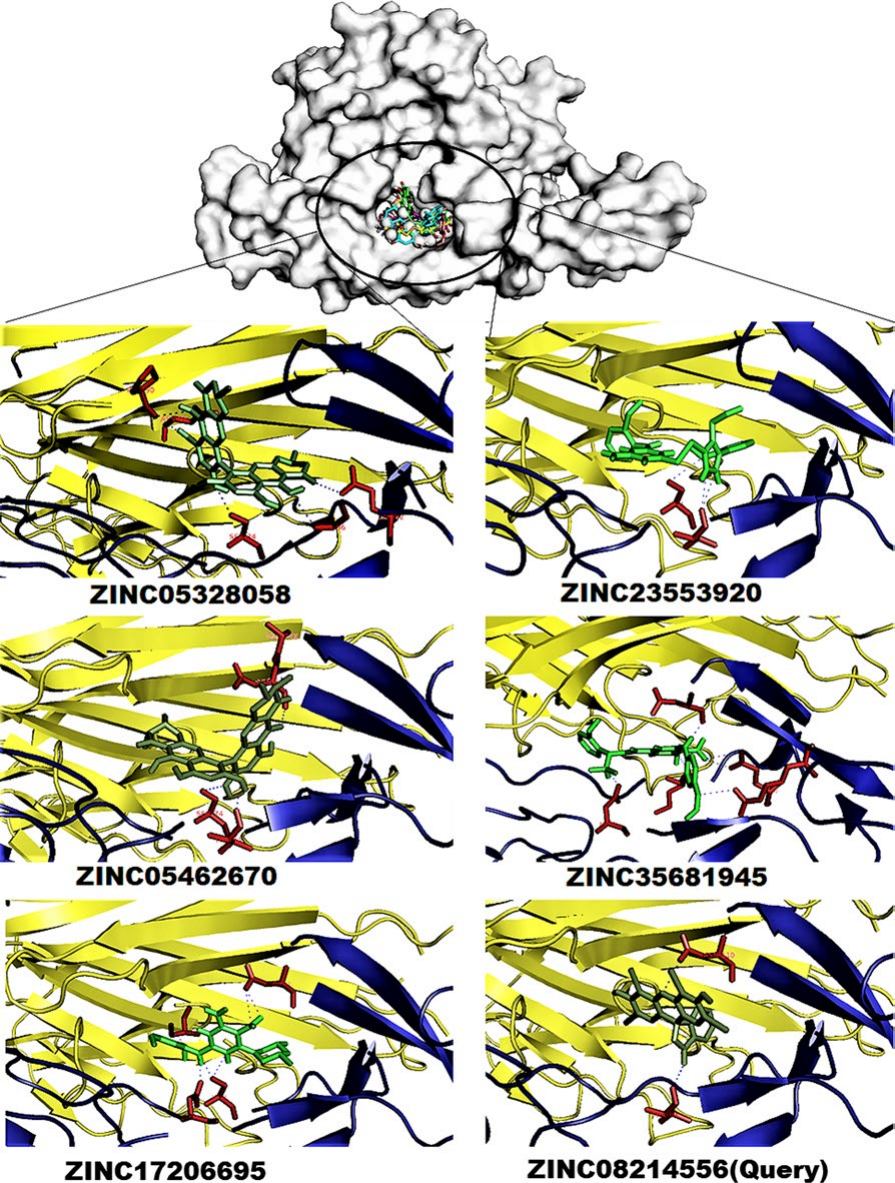
The top five ligand molecular poses of docking protein–ligand interaction analysis of the TNFα-TNFR1 complex along with docking functional residues. The names of these compounds are ZINC05462670, ZINC35681945, ZINC23553920, ZINC05328058, and ZINC17206695

The TNFα-TNFR1 complex inhibitors, ZINC05462670 molecule OH, OH, OC, and OH groups interacted with four hydrogen bonds to the Ser74, Thr94, Glu109 and Asn110 amino acid CO, CO, CO, and CO functional groups with − 10.0 kcal/mol^−1^ binding energy respectively. The ZINC35681945 molecule NH, ON, ON, NH, OC and NH groups interacted with six hydrogen bonds to the Lys75, Gln82, Gln82, Asp93, Arg104 and Asn110 amino acid CO, CO, CN, CO, CN and CO functional groups with − 9.7 kcal/mol^−1^ binding energy. The ZINC23553920 molecule OC, NH, and NH groups interacted with three hydrogen bonds to the Ser74, Thr94, and Thr94 amino acid CO, CO, and CO functional groups with − 9.5 kcal/mol^−1^ binding energy respectively. The ZINC05328058 molecule OH, OC, OH, OH and OH groups interacted with five hydrogen bonds to the Ser74, Gln82, Pro90, Asn92, and Cys96 amino acid CO, CO, CO, CN and CN functional groups with − 8.9 kcal/mol^−1^ binding energy respectively. The ZINC17206695 molecule NH, NH, NH, ON, NO and OC groups interacted with six hydrogen bonds to the Ser74, Ser74, Asn93, Asn110, Asn110 and Ser147 amino acid CO, CO, CO, CN, CN and OC functional groups with − 8.5 kcal/mol^−1^ binding energy respectively. The ZINC08214556 (Query) molecule OC, and OC groups interacted with six hydrogen bonds to the Thr94 and Asn110 amino acid CO, and CN functional groups with − 7.2 kcal/mol^−1^ binding energy respectively (Fig. 6). The functional key residues play a vital role in TNFR1 and TNFα-TNFR1 complex formation and activation of the TNFα signaling pathway in proinflammation.

### Chemical analysis of drug-likeness

All fifteen inhibitors were performed Lipinski “Rule of 5” and “drug-likeness” by MolSoft (https://www.molsoft.com/) and FAF-Drugs4 (http://fafdrugs4.mti.univ-paris-diderot.fr/) tools. The compounds showed Log P ≤ 5, relative molecular mass ≤ 500, range of HBA (hydrogen bond acceptors) ≤ 10, and range of HBD (hydrogen bond donors) ≤ 5 considering the best ligand molecules were used as drug leads for biological activity (Table 2). Lipinski’s Rule of five could be a rule of thumb designed for evaluating the drug likeness, or deciding whether a substance through a particular pharmacologic or biological action that may possibly create it a credible verbally energetic compound in humans. The results showed that fifteen TNFα, TNFR1 and TNFα–TNFR1 complex inhibitors, i.e., ZINC09609430, ZINC49467549, ZINC13113075, ZINC39907639, ZINC25251930, ZINC02968981, ZINC09544246, ZINC58047088, ZINC72021182, ZINC08704414, ZINC05462670, ZINC35681945, ZINC23553920, ZINC05328058 and ZINC17206695 satisfied the Rule of five and drug-likeness.

**Table 2:** TNF-α, TNFR1, TNF-α -TNFR1 complex inhibitors and their molecular properties and drug-likeness predicted by Molsoft and FAF-Drugs4

TNF-α inhibitors						
Molecular Properties and drug-likeness	ZINC09609430	ZINC13113075	ZINC25251930	ZINC39907639	ZINC49467549	307 (default ligand)
Molecular formula	C28 H29 F N4 O3	C22 H20 F N3 O3 S	C25 H22 N6 O	C28 H26 N2 O3	C28 H24 N4 O3 S	–
Molecular weight	488.22	425.12	422.19	438.19	496.16	–
Number of HBA	5	6	4	4	6	–
Number of HBD	1	1	1	0	1	–
MolLogP	4.21	4.10	4.08	6.19 (> 5)	4.83	–
MolLogS	− 7.68 (in Log(moles/L)) 0.01 (in mg/L)	− 6.63 (in Log(moles/L)) 0.10 (in mg/L)	− 5.18 (in Log(moles/L)) 2.78 (in mg/L)	− 6.59 (in Log(moles/L)) 0.11 (in mg/L)	− 5.50 (in Log(moles/L)) 1.59 (in mg/L)	–
MolPSA	60.03 A^2^	60.52 A^2^	54.90 A^2^	33.34 A^2^	70.46 A^2^	–
MolVol	490.82 A^3^	401.38 A^3^	420.54 A^3^	435.86 A^3^	495.76 A^3^	–
Number of stereo centers	1	2	1	0	0	–
Drug-likeness model score	1.06	0.30	0.56	0.81	0.74	–

TNFR1 inhibitors						
Molecular Properties and drug-likeness	ZINC02968981	ZINC08704414	ZINC09544246	ZINC58047088	ZINC72021182	ZINC33832439 (query)
Molecular formula	C24 H18 N6 O3 S	C21 H21 N5 O S	C18 H15 N5 O4 S2	C20 H17 F N5 O2 S	C23 H19 Cl N2 O4	C22 H22 O10
Molecular weight	470.12	391.15	429.06	410.11	422.10	446.12
Number of HBA	7	5	8	5	4	10
Number of HBD	1	1	4	2	3	5
MolLogP	4.10	3.50	1.94	3.31	3.44	0.23
MolLogS	− 6.69 (in Log(moles/L)) 0.10 (in mg/L)	− 4.42 (in Log(moles/L)) 14.86 (in mg/L)	− 6.71 (in Log(moles/L)) 0.08 (in mg/L)	− 4.98 (in Log(moles/L)) 4.30 (in mg/L)	− 5.25 (in Log(moles/L)) 2.36 (in mg/L)	− 5.78 (in Log(moles/L)) 0.74 (in mg/L)
MolPSA	92.53 A^2^	54.37 A^2^	113.69 A^2^	67.36 A^2^	79.03 A^2^	130.22 A^2^
MolVol	417.94 A^3^	371.69 A^3^	367.90 A^3^	387.22 A^3^	397.84 A^3^	411.01 A^3^
Number of stereo centers	0	1	0	0	0	5
Drug-likeness model score	0.34	0.59	0.78	1.05	1.91	0.77

TNF-α–TNFR1 complex inhibitors						
Molecular Properties and drug-likeness	ZINC05328058	ZINC23553920	ZINC17206695	ZINC05462670	ZINC35681945	ZINC08214556 (query)
Molecular formula	C28 H22 O10	C24 H26 N8 O S	C19 H26 N6 O4	C30 H26 O10	C23 H30 N7 O4	C20 H8 I4 O5
Molecular weight	518.12 (> 500)	474.20	402.20	546.15 (> 500)	468.24	835.66 (> 500)
Number of HBA	10	7	6	10	6	5
Number of HBD	6 (> 5)	3	3	6 (> 5)	5	2
MolLogP	4.07	5.02 (> 5)	3.47	5.12 (> 5)	2.53	7.86 (> 5)
MolLogS	− 5.82 (in Log(moles/L)) 0.79 (in mg/L)	− 7.02 (in Log(moles/L)) 0.05 (in mg/L)	− 5.61 (in Log(moles/L)) 0.99 (in mg/L)	− 6.81 (in Log(moles/L)) 0.08 (in mg/L)	− 6.48 (in Log(moles/L)) 0.15 (in mg/L)	− 6.87 (in Log(moles/L)) 0.11 (in mg/L)
MolPSA	137.14 A^2^	92.52 A^2^	104.94 A^2^	138.17 A^2^	115.18 A^2^	58.97 A^2^
MolVol	489.42 A^3^	451.02 A^3^	377.73 A^3^	528.68 A^3^	444.44 A^3^	430.17 A^3^
Number of stereo centers	2	0	2	4	1	0
Drug-likeness model score	− 0.35	0.14	0.54	− 0.69	1.12	0.20

### Prediction of physicochemical descriptors and ADMET parameters

**Fig. 7:**
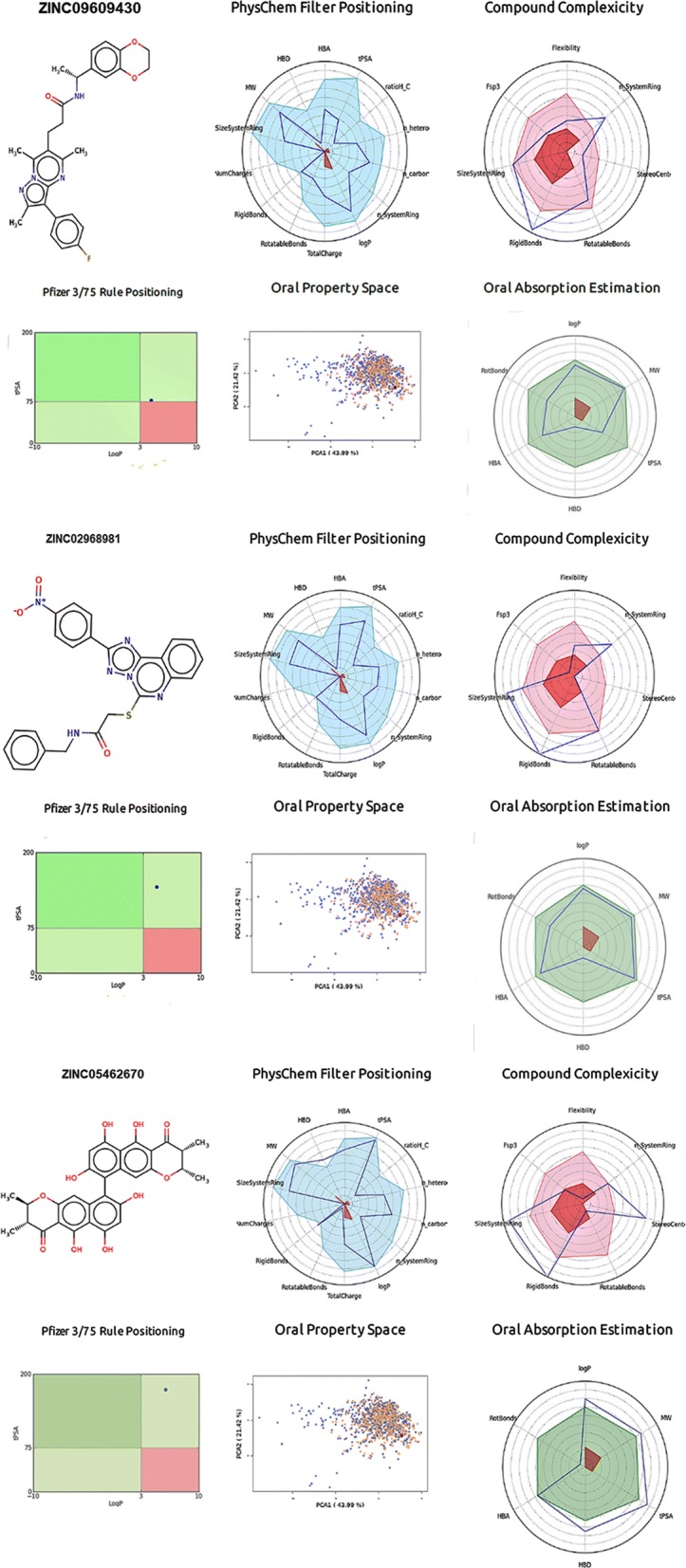
The ADMET properties (2D structure of each ligand atoms, physicochemical filter positioning, compound complexity, oral property space, oral absorption estimation and Pfizer 3/75 rule positioning) of **a** the best TNF-α inhibitor (ZINC09609430), **b** the TNFR1 inhibitor (ZINC02968981), and **c** the TNF-α–TNFR1 complex inhibitor (ZINC05462670)

We analyzed physicochemical descriptors and ADMET parameters by FAF-Drugs4 and SwissADME analysis to find the solubility and permeability of the 15 ligand molecules in order to use them for experimental assays and to reach their site of action in an accurate drug ability. The molecular complexity of the fifteen ligands could be measured by the number of rings and aromatic rings, the fraction of carbons that were sp3 hybridized (Fsp3), or the number of stereocenter properties and ADMET properties, which were all computed by FAF-Drugs4 (Additional file 1: Figs. S1 and S2). The TNFα, TNF1 and TNFα–TNF1 complex of best compounds (ZINC09609430, ZINC02968981 and ZINC05462670) FAF-Drugs4 results are presented in Fig. 7. The in silico ligand toxicity and biological property predictions are faster and more reliable approaches to take before further exploring experimental authentications such as in vitro and in vivo tests. All fifteen inhibitors were screened with the SwissADME server. The results revealed that all 15 ligands were safe and passed the lipophilicity, water solubility, pharmacokinetics, drug likeness and medicinal chemistry properties. All fifteen TNFα, TNF1 and TNFα-TNF1 complex inhibitor molecules obeyed the Lipinski rule of five and ADMET properties with biologically possible activity (Additional file 1: Tables S1, S2, S3). Therefore, these TNFα, TNF1 and TNFα-TNF1 complex inhibitors are most appropriate for additional drug discovery approaches to drug discovery.

## Discussion

The present study screened for novel small inhibitors that can specifically inhibit TNFα–TNFR interaction and downstream signaling. We identified the key amino acid residues involved in the interactions of the TNFα and TNFR1 proteins. We screened the lead compound hits for TNFα, TNFR1 and the TNFα-TNFR1 complex from the Zinc library using structure-based pharmacophore modeling, virtual screening, and molecular docking along with in silico ADMET analysis. The identified novel small inhibitors can potentially be utilized for anti-inflammatory agents to treat relevant disorders.

The pharmacophore features are the key elements to screen for the best, potent small molecules binding to target proteins from publicly available databases. Pharmacophore-based approaches were widely used in virtual screening, de novo design and other applications such as lead optimization and multitarget drug design [30]. For TNFα, TNFR1 and the TNFα–TNFR1 complex, we used six pharmacophore features with the default ligand (307), five pharmacophore features with a Physcion-8-glucoside ligand, and five pharmacophore features with an Erythrosine B ligand respectively. Based on these pharmacophore features, we identified 39, 37 and 45 best hits from the Zinc database. Molecular docking results revealed that the aforementioned hits exactly docked into the active site of TNFα and TNFR1. Protein–ligand interactions suggested that the functional groups (residues) mimic the binding of hits and fit well into the active domain of TNFα, TNFR1 and the TNFα–TNFR1 complex. In particular, the Ile58, Leu120, Gly121, Tyr515, Glu56, Ser57, Ser59, Cys70, Cys73, Ser74, Lys75, Arg77, Gln82, Cys96, Arg104, Tyr106, and Asn110 residues are critical for the inhibitory interaction between TNFα, TNFR1 and the TNFα–TNFR1 complex. These key residues are located in the TNFR1 binding site of the TNFα protein. The in silico ADMET results revealed that all the top five of TNFα, TNFR1 and the TNFα–TNFR1 complex’s inhibitors are virtually safe and active.

TNF is a cytokine protein expressed by activated monocytes/macrophages (including central nervous system [CNS] microglia), activated NK (Natural killer) and T cells, and by a diverse array of non-immune cells such as endothelial cells and fibroblasts [31]. TNFα is produced in two forms, soluble TNFα (sTNFα) and membrane-bound TNFα (tmTNFα). The soluble form of TNFα is created from the tmTNFα extracellular domain by the matrix metalloproteinase TNFα converting enzyme (TACE) [32]. Membrane-bound TNFα is able to serve as a ligand binding to TNFR or as a receptor mediating the transfer of external signals back to the cell which has exprimed it on its surface [33]. Both cytokine forms, i.e. soluble and membrane bound are active as homotrimers with a characteristic cone-shape. The five best TNFα inhibitors interact with the TNFα homodimer and inhibit the active form of homotrimers. Oanh et al. [34] reported that the triterpene saponins had a good binding affinity with protein TNFα and were docked to the pore at the top of the bell or cone shaped TNFα trimer. Mehreen et al. [35] also reported that the novel small molecules interacted with TNFα trimer. The docking results are in agreement with the findings from the literatures. Our in silico method identified that TNFα inhibitors may disrupt the trimer formation of TNFα.

The trimer form of TNF binds to TNFR1, activates the downstream signaling, and predominantly promotes inflammation and tissue degeneration [36]. The five best TNFR1 inhibitors interacted with the TNFα binding site of TNFR1 and inhibited the TNF/TNFR1 signaling pathway. Chen et al. [23] also reported that small molecules that directly bind to TNFα or TNFR1, inhibit the interaction between TNFα and TNFR1, and/or regulate related signaling pathways. Fischer et al. [37] reported the sTNF/TNFR1 signaling as a new therapeutic target pathway. Recent researchers have focused primarily on identifying small molecules that directly bind to TNFα or TNFR1 [38], inhibit the binding of the TNFα and TNFR1 [39] and regulate related signal pathways [40]. The TNFR1 docking results are consistent with the results by other investigators. Identifying potential inhibitors of TNFα, TNFR1 and TNFα–TNFR1 complex and its analogues is thus an attractive strategy for treating inflammatory diseases, such as in central nervous system (i.e. brain and retina). Using our established cheminformatics pipeline, we identified 15 inhibitors. These novel inhibitors are worthy of further assessment for safety and efficacy in vitro and in vivo.

## Conclusion

In the present work, we established a pharmacophore model to recognize vitally assorted lead hits for TNFα, TNFR1 and the TNFα-TNFR1 complex. The recognized hit compounds were utilized to create novel, strong inhibitors for the targets, and further assessed by docking and in silico ADMET studies. Fifteen lead compounds satisfied all the criteria and serve as novel, structurally diverse inhibitors for TNFα, TNFR1 and the TNFα–TNFR1 complex.

## Additional file

**Additional file 1: Fig. S1.** FAF-Drugs4 ADME results for the TNF-α best ligand molecules and their respective properties such as: 2D structure of each ligand atoms, physicochemical filter positioning, compound complexity, oral property space, oral absorption estimation and Pfizer 3/75 rule positioning. **Fig. S2.** FAF-Drugs4 ADME results for the TNFR1 best ligand molecules and their respective properties such as: 2D structure of each ligand atoms, physicochemical filter positioning, compound complexity, oral property space, oral absorption estimation and Pfizer 3/75 rule positioning. **Fig. S3.** FAF-Drugs4 ADME results for the TNF-α–TNFR1 complex best ligand molecules and their respective properties such as: 2D structure of each ligand atoms, physicochemical filter positioning, compound complexity, oral property space, oral absorption estimation and Pfizer 3/75 rule positioning. **Table S1.** TNF-α and its inhibitors to compute physicochemical descriptors as well as to predict ADME parameters, pharmacokinetic properties, druglike nature and medicinal chemistry friendliness properties predicted by SwissADME tool. **Table S2.** TNFR1 and its inhibitors to compute physicochemical descriptors as well as to predict ADME parameters, pharmacokinetic properties, druglike nature and medicinal chemistry friendliness properties predicted by SwissADME tool. **Table S3.** TNF-α -TNFR1 complex and its inhibitors to compute physicochemical descriptors as well as to predict ADME parameters, pharmacokinetic properties, druglike nature and medicinal chemistry friendliness properties predicted by SwissADME tool.

## Data Availability

All data generated and analyzed during this study are included in this published article and its additional information. Raw in silico datasets are available from the corresponding author on reasonable request.

## Abbreviations

TNFα: tumor necrosis factor alpha
TNFR1: tumor necrosis factor receptor1
ADMET: absorption, distribution, metabolism, excretion and toxicity
IL-1β: interleukin 1 beta
IFN-γ: interferon gamma
VEGF: vascular endothelial growth factor
RPE: retinal pigment epithelial
CASTp: computed atlas of surface topography of proteins
PDB: protein data bank
HBA: hydrogen bond acceptors
HBD: hydrogen bond donor
BBB: blood brain barrier
PAINS: Pan-Assay Interference Compounds
UFF: universal force field
IUPAC: International Union of Pure and Applied Chemistry)
RMSD: root mean square deviation
MW: molecular weight
EF: enrichment factor
HR: hit rate
SMILES: simplified molecular-input line-entry system
CNS: central nervous system
NK: natural killer
