# Supplementary material for: Identification of novel inhibitors for TNFα, TNFR1 and TNFα-TNFR1 complex using pharmacophore-based approaches
Source: J Transl Med. 2019 Jul 2;17:215. doi: 10.1186/s12967-019-1965-5 (PMC6604280; doi:10.1186/s12967-019-1965-5)
Supplement: Supplementary file 1 — Additional file 1: Fig. S1. FAF-Drugs4 ADME results for the TNF-α best ligand molecules and their respective properties such as: 2D structure of each ligand atoms, physicochemical filter positioning, compound complexity, oral property space, oral absorption estimation and Pfizer 3/75 rule positioning. Fig. S2. FAF-Drugs4 ADME results for the TNFR1 best ligand molecules and their respective properties such as: 2D structure of each ligand atoms, physicochemical filter positioning, compound complexity, oral property space, oral absorption estimation and Pfizer 3/75 rule positioning. Fig. S3. FAF-Drugs4 ADME results for the TNF-α–TNFR1 complex best ligand molecules and their respective properties such as: 2D structure of each ligand atoms, physicochemical filter positioning, compound complexity, oral property space, oral absorption estimation and Pfizer 3/75 rule positioning. Table S1. TNF-α and its inhibitors to compute physicochemical descriptors as well as to predict ADME parameters, pharmacokinetic properties, druglike nature and medicinal chemistry friendliness properties predicted by SwissADME tool. Table S2. TNFR1 and its inhibitors to compute physicochemical descriptors as well as to predict ADME parameters, pharmacokinetic properties, druglike nature and medicinal chemistry friendliness properties predicted by SwissADME tool. Table S3. TNF-α -TNFR1 complex and its inhibitors to compute physicochemical descriptors as well as to predict ADME parameters, pharmacokinetic properties, druglike nature and medicinal chemistry friendliness properties predicted by SwissADME tool. [file 12967_2019_1965_MOESM1_ESM.docx]

**Additional files**

**Figures**

**Figure S1**


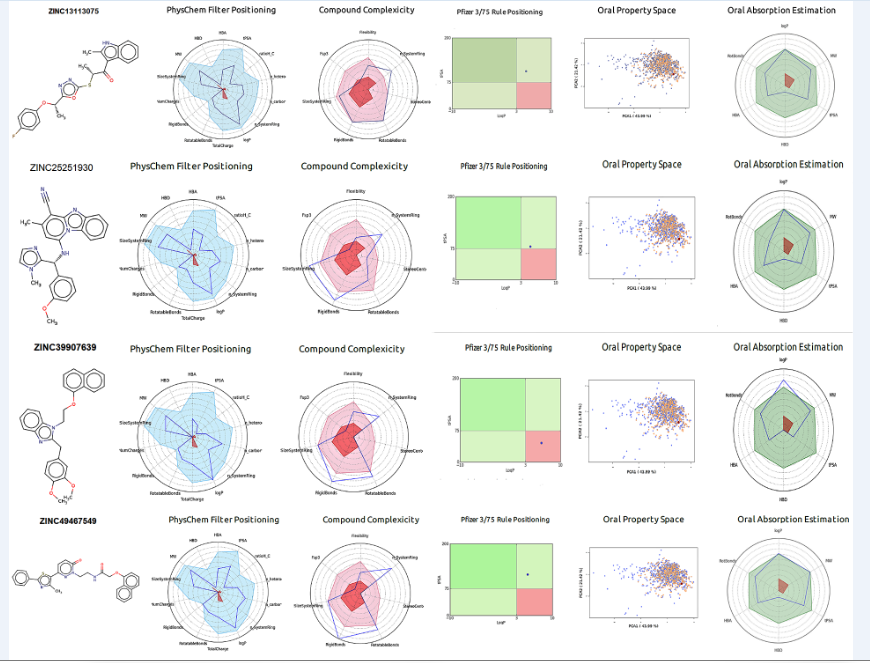


**Figure S2**

**
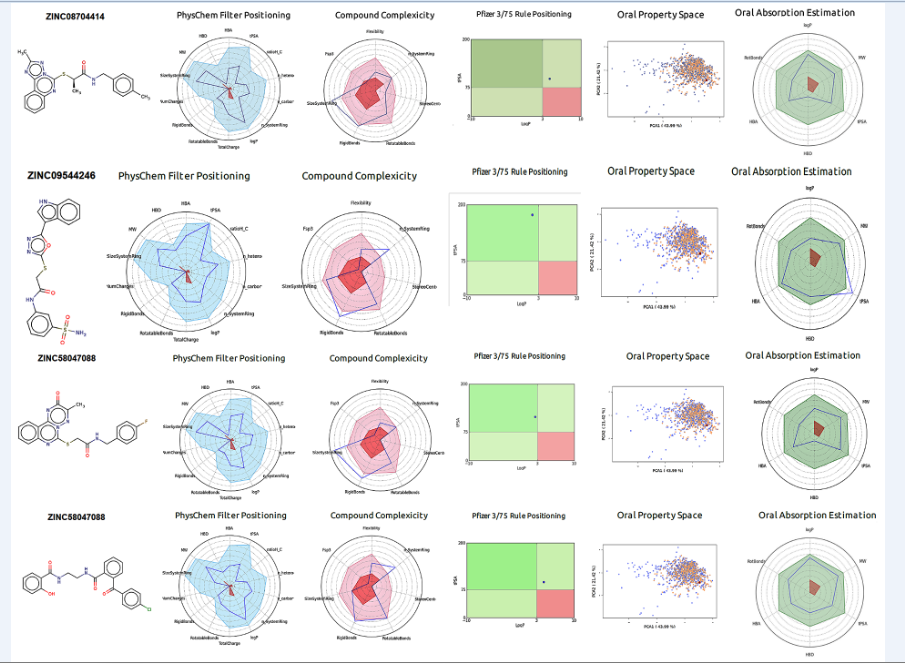
**

**Figure S3**

**
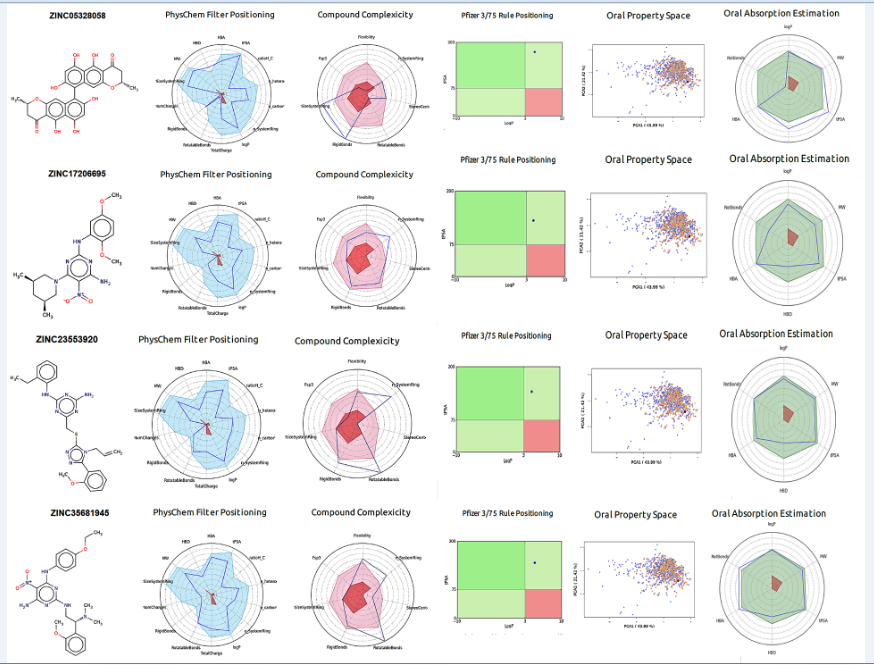
**

**Tables**

**Table S1:** TNF-α and its inhibitors to compute physicochemical descriptors as well as to predict ADME parameters, pharmacokinetic properties, druglike nature and medicinal chemistry friendliness properties predicted by SwissADME tool.

| **SwissADME** | **ZINC09609430** | **ZINC49467549** | **ZINC13113075** | **ZINC39907639** | **ZINC25251930** | **307 (query)** |
| --- | --- | --- | --- | --- | --- | --- |
| **Physicochemical Properties** | | | | | | |
| Formula | C28H29FN4O3 | C28H24N4O3S | C22H20FN3O3S | C28H26N2O3 | C25H22N6O |  |
| Molecular weight | 488.55 g/mol | 496.58 g/mol | 425.48 g/mol | 438.52 g/mol | 422.48 g/mol | 0.00 g/mol |
| Num. heavy atoms | 36 | 36 | 30 | 33 | 32 | 0 |
| Num. arom. heavy atoms | 21 | 27 | 20 | 25 | 24 | 0 |
| Fraction Csp3 | 0.32 | 0.14 | 0.23 | 0.18 | 0.16 | 0.16 |
| Num. rotatable bonds | 7 | 9 | 7 | 8 | 5 | 0 |
| Num. H-bond acceptors | 6 | 5 | 6 | 4 | 4 | 4 |
| Num. H-bond donors | 1 | 1 | 1 | 0 | 1 | 1 |
| Molar Refractivity | 135.83 | 142.21 | 113.11 | 131.76 | 124.20 | 0.00 |
| TPSA | 77.75 Å² | 114.35 Å² | 106.31 Å² | 45.51 Å² | 80.17 Å² | 0.00 Å² |
| **Lipophilicity** | | | | | | |
| Log *P*_o/w_ (iLOGP) | 4.26 | 3.79 | 3.63 | 3.71 | 3.29 | 3.29 |
| Log *P*_o/w_ (XLOGP3) | 4.37 | 5.13 | 4.94 | 6.19 | 4.06 | 4.06 |
| Log *P*_o/w_ (WLOGP) | 5.14 | 4.69 | 5.60 | 5.88 | 4.10 | 4.10 |
| Log *P*_o/w_ (MLOGP) | 3.58 | 2.81 | 2.92 | 3.78 | 1.93 | 1.93 |
| Log *P*_o/w_ (SILICOS-IT) | 5.99 | 6.03 | 5.54 | 5.82 | 3.28 | 3.28 |
| Consensus Log *P*_o/w_ | 4.67 | 4.49 | 4.53 | 5.07 | 3.33 | 3.33 |
| **Water Solubility** | | | | | | |
| Log *S* (ESOL) | -5.59 | -6.11 | -5.62 | -6.49 | -5.24 | -5.24 |
| Solubility | 1.25e-03 mg/ml; 2.56e-06 mol/l | 3.84e-04 mg/ml; 7.73e-07 mol/l | 1.02e-03 mg/ml; 2.39e-06 mol/l | 1.42e-04 mg/ml; 3.23e-07 mol/l | 2.42e-03 mg/ml; 5.73e-06 mol/l | 2.42e-03 mg/ml; 5.73e-06 mol/l |
| Class | Moderately soluble | Poorly soluble | Moderately soluble | Poorly soluble | Moderately soluble | Moderately soluble |
| Log *S* (Ali) | -5.72 | -7.28 | -6.91 | -6.93 | -5.45 | -5.45 |
| Solubility | 9.34e-04 mg/ml; 1.91e-06 mol/l | 2.63e-05 mg/ml; 5.30e-08 mol/l | 5.24e-05 mg/ml; 1.23e-07 mol/l | 5.15e-05 mg/ml; 1.17e-07 mol/l | 1.51e-03 mg/ml; 3.57e-06 mol/l | 1.51e-03 mg/ml; 3.57e-06 mol/l |
| Class | Moderately soluble | Poorly soluble | Poorly soluble | Poorly soluble | Moderately soluble | Moderately soluble |
| Log *S* (SILICOS-IT) | -9.30 | -10.03 | -8.02 | -9.89 | -7.80 | -7.80 |
| Solubility | 2.45e-07 mg/ml; 5.01e-10 mol/l | 4.68e-08 mg/ml ; 9.42e-11 mol/l | 4.04e-06 mg/ml ; 9.48e-09 mol/l | 5.62e-08 mg/ml ; 1.28e-10 mol/l | 6.73e-06 mg/ml ; 1.59e-08 mol/l | 6.73e-06 mg/ml ; 1.59e-08 mol/l |
| Class | Poorly soluble | Insoluble | Poorly soluble | Poorly soluble | Poorly soluble | Poorly soluble |
| **Pharmacokinetics** | | | | | | |
| GI absorption | High | High | Low | High | High | High |
| BBB permeant | No | No | No | Yes | No | No |
| P-gp substrate | Yes | Yes | Yes | Yes | Yes | Yes |
| CYP1A2 inhibitor | No | No | Yes | Yes | No | No |
| CYP2C19 inhibitor | Yes | Yes | Yes | Yes | Yes | Yes |
| CYP2C9 inhibitor | Yes | Yes | Yes | Yes | Yes | Yes |
| CYP2D6 inhibitor | Yes | No | Yes | Yes | Yes | Yes |
| CYP3A4 inhibitor | Yes | Yes | Yes | Yes | Yes | Yes |
| Log *K*_p_ (skin permeation) | -6.18 cm/s | -5.69 cm/s | -5.39 cm/s | -4.58 cm/s | -5.99 cm/s | -5.99 cm/s |
| **Drug likeness** | | | | | | |
| Lipinski | Yes; 0 violation | Yes; 0 violation | Yes; 0 violation | Yes; 0 violation | Yes; 0 violation | Yes; 0 violation |
| Ghose | No; 2 violations: MW>480, MR>130 | No; 2 violations: MW>480, MR>130 | Yes | No; 2 violations: WLOGP>5.6, MR>130 | Yes | Yes |
| Veber | Yes | Yes | Yes | Yes | Yes | Yes |
| Egan | Yes | Yes | Yes | Yes | Yes | Yes |
| Muegge | Yes | No; 1 violation: XLOGP3>5 | Yes | No; 1 violation: XLOGP3>5 | Yes | Yes |
| Bioavailability Score | 0.55 | 0.55 | 0.55 | 0.55 | 0.55 | 0.55 |
| **Medicinal Chemistry** | | | | | | |
| PAINS | 0 alert | 0 alert | 0 alert | 0 alert | 0 alert | 0 alert |
| Brenk | 0 alert | 0 alert | 0 alert | 0 alert | 0 alert | 0 alert |
| Leadlikeness | No; 2 violations: MW>350, XLOGP3>3.5 | No; 3 violations: MW>350, Rotors>7, XLOGP3>3.5 | No; 2 violations: MW>350, XLOGP3>3.5 | No; 3 violations: MW>350, Rotors>7, XLOGP3>3.5 | No; 2 violations: MW>350, XLOGP3>3.5 | No; 2 violations: MW>350, XLOGP3>3.5 |
| Synthetic accessibility | 4.32 | 4.05 | 4.21 | 3.20 | 3.72 | 3.72 |

**Table S2:** TNFR1 and its inhibitors to compute physicochemical descriptors as well as to predict ADME parameters, pharmacokinetic properties, druglike nature and medicinal chemistry friendliness properties predicted by SwissADME tool.

| **SwissADME** | **ZINC02968981** | **ZINC09544246** | **ZINC58047088** | **ZINC72021182** | **ZINC08704414** | **ZINC33832439(Query)** |
| --- | --- | --- | --- | --- | --- | --- |
| **Physicochemical Properties** | | | | | | |
| Formula | C24H18N6O3S | C18H15N5O4S2 | C20H16FN5O2S | C23H19ClN2O4 | C21H21N5OS | C22H22O10 |
| Molecular weight | 470.50 g/mol | 429.47 g/mol | 409.44 g/mol | 422.86 g/mol | 391.49 g/mo | 446.40 g/mol |
| Num. heavy atoms | 34 | 29 | 29 | 30 | 28 | 32 |
| Num. arom. heavy atoms | 25 | 20 | 20 | 18 | 19 | 12 |
| Fraction Csp3 | 0.08 | 0.06 | 0.15 | 0.09 | 0.24 | 0.36 |
| Num. rotatable bonds | 8 | 7 | 6 | 9 | 6 | 4 |
| Num. H-bond acceptors | 6 | 7 | 6 | 4 | 4 | 10 |
| Num. H-bond donors | 1 | 3 | 1 | 3 | 1 | 5 |
| Molar Refractivity | 131.37 | 108.14 | 108.31 | 113.67 | 111.85 | 107.38 |
| TPSA | 143.30 Å² | 177.65 Å² | 114.55 Å² | 95.50 Å² | 97.48 Å² | 162.98 Å² |
| **Lipophilicity** | | | | | | |
| Log *P*_o/w_ (iLOGP) | 3.11 | 1.59 | 2.96 | 2.90 | 3.33 | 1.23 |
| Log *P*_o/w_ (XLOGP3) | 4.61 | 1.85 | 2.54 | 4.34 | 4.29 | 1.24 |
| Log *P*_o/w_ (WLOGP) | 4.11 | 3.49 | 2.76 | 3.44 | 3.54 | -0.34 |
| Log *P*_o/w_ (MLOGP) | 3.23 | 0.89 | 3.34 | 2.79 | 3.68 | -1.55 |
| Log *P*_o/w_ (SILICOS-IT) | 1.66 | 1.58 | 3.27 | 4.21 | 3.52 | 0.99 |
| Consensus Log *P*_o/w_ | 3.34 | 1.88 | 2.98 | 3.53 | 3.67 | 0.32 |
| **Water Solubility** | | | | | | |
| Log *S* (ESOL) | -5.68 | -3.72 | -4.09 | -5.05 | -5.08 | -3.40 |
| Solubility | 9.89e-04 mg/ml; 2.10e-06 mol/l | 8.25e-02 mg/ml; 1.92e-04 mol/l | 3.30e-02 mg/ml; 8.07e-05 mol/l | 3.80e-03 mg/ml ; 9.00e-06 mol/l | 3.29e-03 mg/ml ; 8.39e-06 mol/l | 1.77e-01 mg/ml ; 3.96e-04 mol/l |
| Class | Moderately soluble | Soluble | Moderately soluble | Moderately soluble | Moderately soluble | Soluble |
| Log *S* (Ali) | -7.34 | -5.20 | -4.59 | -6.06 | -6.05 | -4.26 |
| Solubility | 2.13e-05 mg/ml; 4.53e-08 mol/l | 2.70e-03 mg/ml; 6.29e-06 mol/l | 1.05e-02 mg/ml ; 2.56e-05 mol/l | 3.68e-04 mg/ml ; 8.70e-07 mol/l | 3.49e-04 mg/ml ; 8.91e-07 mol/l | 2.45e-02 mg/ml ; 5.49e-05 mol/l |
| Class | Poorly soluble | Moderately soluble | Moderately soluble | Poorly soluble | Poorly soluble | Moderately soluble |
| Log *S* (SILICOS-IT) | -8.21 | -6.76 | -7.36 | -8.13 | -7.19 | -2.88 |
| Solubility | 2.93e-06 mg/ml; 6.24e-09 mol/ | 7.39e-05 mg/ml; 1.72e-07 mol/l | 1.79e-05 mg/ml ; 4.38e-08 mol/l | 3.11e-06 mg/ml ; 7.36e-09 mol/l | 2.53e-05 mg/ml ; 6.47e-08 mol/l | 5.83e-01 mg/ml ; 1.31e-03 mol/l |
| Class | Poorly soluble | Poorly soluble | Poorly soluble | Poorly soluble | Poorly soluble | Soluble |
| **Pharmacokinetics** | | | | | | |
| GI absorption | Low | Low | High | High | High | Low |
| BBB permeant | No | No | No | No | No | No |
| P-gp substrate | No | No | No | No | No | Yes |
| CYP1A2 inhibitor | Yes | No | Yes | Yes | No | No |
| CYP2C19 inhibitor | Yes | Yes | No | Yes | Yes | No |
| CYP2C9 inhibitor | Yes | No | Yes | Yes | Yes | No |
| CYP2D6 inhibitor | No | No | No | Yes | Yes | No |
| CYP3A4 inhibitor | Yes | Yes | Yes | Yes | Yes | No |
| Log *K*_p_ (skin permeation) | -5.90 cm/s | -7.61 cm/s | -6.99 cm/s | -5.80 cm/s | -5.64 cm/s | -8.14 cm/s |
| **Druglikeness** | | | | | | |
| Lipinski | Yes; 0 violation | Yes; 0 violation | Yes; 0 violation | Yes; 0 violation | Yes; 0 violation | Yes; 0 violation |
| Ghose | No; 1 violation: MR>130 | Yes | Yes | Yes | Yes | Yes |
| Veber | No; 1 violation: TPSA>140 | No; 1 violation: TPSA>140 | Yes | Yes | Yes | No; 1 violation: TPSA>140 |
| Egan | No; 1 violation: TPSA>131.6 | No; 1 violation: TPSA>131.6 | Yes | Yes | Yes | No; 1 violation: TPSA>131.6 |
| Muegge | Yes | No; 1 violation: TPSA>150 | Yes | Yes | Yes | No; 1 violation: TPSA>150 |
| Bioavailability Score | 0.55 | 0.55 | 0.55 | 0.55 | 0.55 | 0.55 |
| **Medicinal Chemistry** | | | | | | |
| PAINS | 0 alert | 0 alert | 0 alert | 0 alert | 0 alert | 1 alert: quinone_A |
| Brenk | 2 alerts: nitro_group, oxygen-nitrogen_single_bond | 0 alert | 1 alert: polycyclic_aromatic_hydrocarbon_3 | 0 alert | 0 alert | 0 alert |
| Lead likeness | No; 3 violations: MW>350, Rotors>7, XLOGP3>3.5 | No; 1 violation: MW>350 | No; 1 violation: MW>350 | No; 3 violations: MW>350, Rotors>7, XLOGP3>3.5 | No; 2 violations: MW>350, XLOGP3>3.5 | No; 1 violation: MW>350 |
| Synthetic accessibility | 3.33 | 3.37 | 3.03 | 2.67 | 3.42 | 5.13 |

**Table S3:** TNF-α -TNFR1 complex and its inhibitors to compute physicochemical descriptors as well as to predict ADME parameters, pharmacokinetic properties, druglike nature and medicinal chemistry friendliness properties predicted by SwissADME tool.

| **SwissADME** | **ZINC05462670** | **ZINC23553920** | **ZINC17206695** | **ZINC05328058** | **ZINC35681945** | **ZINC08214556(Query)** |
| --- | --- | --- | --- | --- | --- | --- |
| **Physicochemical Properties** | | | | | | |
| Formula | C30H26O10 | C24H26N8OS | C19H26N6O4 | C28H22O10 | C23H30N7O4 | C20H8I4O5 |
| Molecular weight | 546.52 g/mol | 474.58 g/mol | 402.45 g/mol | 518.47 g/mol | 468.53 g/mol | 835.89 g/mol |
| Num. heavy atoms | 0.27 | 34 | 29 | 0.21 | 34 | 0.05 |
| Num. arom. heavy atoms | 40 | 23 | 12 | 38 | 18 | 29 |
| Fraction Csp3 | 20 | 0.21 | 0.47 | 20 | 0.30 | 18 |
| Num. rotatable bonds | 1 | 10 | 6 | 1 | 11 | 0 |
| Num. H-bond acceptors | 10 | 6 | 6 | 10 | 6 | 5 |
| Num. H-bond donors | 6 | 2 | 2 | 6 | 4 | 2 |
| Molar Refractivity | 147.40 | 135.67 | 116.85 | 137.79 | 134.96 | 139.61 |
| TPSA | 173.98 Å² | 141.96 Å² | 131.35 Å² | 173.98 Å² | 144.58 Å² | 75.99 Å² |
| **Lipophilicity** | | | | | | |
| Log *P*_o/w_ (iLOGP) | 3.80 | 3.88 | 3.51 | 3.49 | 2.57 | 3.44 |
| Log *P*_o/w_ (XLOGP3) | 5.94 | 4.55 | 4.23 | 4.79 | 4.86 | 6.02 |
| Log *P*_o/w_ (WLOGP) | 5.09 | 4.36 | 2.84 | 4.60 | 1.91 | 5.98 |
| Log *P*_o/w_ (MLOGP) | 0.65 | 2.70 | 1.03 | 0.26 | -0.92 | 5.50 |
| Log *P*_o/w_ (SILICOS-IT) | 3.92 | 3.50 | -0.45 | 3.67 | 0.18 | 7.21 |
| Consensus Log *P*_o/w_ | 3.88 | 3.80 | 2.23 | 3.36 | 1.72 | 5.63 |
| **Water Solubility** | | | | | | |
| Log *S* (ESOL) | -7.27 | -5.49 | -4.91 | -6.40 | \| -5.47 \| \| --- \| \|  \| | -9.27 |
| Solubility | 2.90e-05 mg/ml; 5.31e-08 mol/l | 1.54e-03 mg/ml; 3.24e-06 mol/l | 4.95e-03 mg/ml ; 1.23e-05 mol/l | 2.08e-04 mg/ml; 4.02e-07 mol/l | 1.58e-03 mg/ml ; 3.37e-06 mol/l | 4.44e-07 mg/ml; 5.32e-10 mol/l |
| Class | Poorly soluble | Moderately soluble | Moderately soluble | Poorly soluble | Moderately soluble | Poorly soluble |
| Log *S* (Ali) | -9.37 | -7.25 | -6.70 | -8.18 | -7.63 | -7.39 |
| Solubility | 2.34e-07 mg/ml; 4.28e-10 mol/l | 2.64e-05 mg/ml ; 5.57e-08 mol/l | 8.05e-05 mg/ml ; 2.00e-07 mol/l | 3.46e-06 mg/ml; 6.68e-09 mol/l | 1.10e-05 mg/ml ; 2.34e-08 mol/l | 3.37e-05 mg/ml; 4.04e-08 mol/l |
| Class | Poorly soluble | Poorly soluble | Poorly soluble | Poorly soluble | Poorly soluble | Poorly soluble |
| Log *S* (SILICOS-IT) | -6.13 | -8.30 | -4.46 | -5.85 | -6.89 | -9.32 |
| Solubility | 4.06e-04 mg/ml; 7.43e-07 mol/l | 2.38e-06 mg/ml ; 5.01e-09 mol/l | 1.41e-02 mg/ml ; 3.50e-05 mol/l | 7.34e-04 mg/ml; 1.42e-06 mol/l | 5.99e-05 mg/ml ; 1.28e-07 mol/l | 3.96e-07 mg/ml; 4.73e-10 mol/l |
| Class | Poorly soluble | Poorly soluble | Moderately soluble | Moderately soluble | Poorly soluble | Poorly soluble |
| **Pharmacokinetics** | | | | | | |
| GI absorption | Low | Low | High | Low | Low | High |
| BBB permeant | No | No | No | No | No | No |
| P-gp substrate | No | No | No | No | No | No |
| CYP1A2 inhibitor | No | No | No | No | No | No |
| CYP2C19 inhibitor | No | Yes | Yes | No | Yes | No |
| CYP2C9 inhibitor | Yes | Yes | Yes | Yes | Yes | No |
| CYP2D6 inhibitor | No | No | Yes | No | No | No |
| CYP3A4 inhibitor | No | Yes | No | No | Yes | No |
| Log *K*_p_ (skin permeation) | -5.42 cm/s | -5.96 cm/s | -5.75 cm/s | -6.06 cm/s | -5.71 cm/s | -7.12 cm/s |
| **Druglikeness** | | | | | | |
| Lipinski | No; 2 violations: MW>500, NHorOH>5 | Yes; 0 violation | Yes; 0 violation | No; 2 violations: MW>500, NHorOH>5 | Yes; 1 violation: NorO>10 | No; 2 violations: MW>500, MLOGP>4.15 |
| Ghose | No; 2 violations: MW>480, MR>130 | No; 1 violation: MR>130 | Yes | No; 2 violations: MW>480, MR>130 | No; 1 violation: MR>130 | No; 3 violations: MW>480, WLOGP>5.6, MR>130 |
| Veber | No; 1 violation: TPSA>140 | No; 1 violation: TPSA>140 | Yes | No; 1 violation: TPSA>140 | No; 2 violations: Rotors>10, TPSA>140 | Yes |
| Egan | No; 1 violation: TPSA>131.6 | No; 1 violation: TPSA>131.6 | Yes | No; 1 violation: TPSA>131.6 | No; 1 violation: TPSA>131.6 | No; 1 violation: WLOGP>5.88 |
| Muegge | No; 3 violations: XLOGP3>5, TPSA>150, H-don>5 | Yes | Yes | No; 2 violations: TPSA>150, H-don>5 | Yes | No; 2 violations: MW>600, XLOGP3>5 |
| Bioavailability Score | 0.17 | 0.55 | 0.55 | 0.17 | 0.55 | 0.17 |
| **Medicinal Chemistry** | | | | | | |
| PAINS | 0 alert | 0 alert | 0 alert | 0 alert | 0 alert | 0 alert |
| Brenk | 0 alert | 1 alert: isolated_alkene | 2 alerts: nitro_group, oxygen-nitrogen_single_bond | 0 alert | 2 alerts: nitro_group, oxygen-nitrogen_single_bond | 1 alert: iodine |
| Leadlikeness | No; 2 violations: MW>350, XLOGP3>3.5 | No; 3 violations: MW>350, Rotors>7, XLOGP3>3.5 | No; 2 violations: MW>350, XLOGP3>3.5 | No; 2 violations: MW>350, XLOGP3>3.5 | No; 3 violations: MW>350, Rotors>7, XLOGP3>3.5 | No; 2 violations: MW>350, XLOGP3>3.5 |
| Synthetic accessibility | 4.93 | 3.92 | 4.19 | 4.33 | 4.27 | 4.16 |
